# Impact of Climate Change on Ambient Ozone Level and Mortality in Southeastern United States

**DOI:** 10.3390/ijerph7072866

**Published:** 2010-07-14

**Authors:** Howard H. Chang, Jingwen Zhou, Montserrat Fuentes

**Affiliations:** 1 Statistical and Applied Mathematical Sciences Institute, 19 T.W. Alexander Drive Research Triangle Park, NC 27709, USA; 2 Statistics Department, North Carolina State University, Raleigh, NC 27695, USA; E-Mails: jzhou3@ncsu.edu (J.Z.); montse_fuentes@ncsu.edu (M.F.)

**Keywords:** climate change, health impact, ground-level ozone

## Abstract

There is a growing interest in quantifying the health impacts of climate change. This paper examines the risks of future ozone levels on non-accidental mortality across 19 urban communities in Southeastern United States. We present a modeling framework that integrates data from climate model outputs, historical meteorology and ozone observations, and a health surveillance database. We first modeled present-day relationships between observed maximum daily 8-hour average ozone concentrations and meteorology measured during the year 2000. Future ozone concentrations for the period 2041 to 2050 were then projected using calibrated climate model output data from the North American Regional Climate Change Assessment Program. Daily community-level mortality counts for the period 1987 to 2000 were obtained from the National Mortality, Morbidity and Air Pollution Study. Controlling for temperature, dew-point temperature, and seasonality, relative risks associated with short-term exposure to ambient ozone during the summer months were estimated using a multi-site time series design. We estimated an increase of 0.43 ppb (95% PI: 0.14–0.75) in average ozone concentration during the 2040’s compared to 2000 due to climate change alone. This corresponds to a 0.01% increase in mortality rate and 45.2 (95% PI: 3.26–87.1) premature deaths in the study communities attributable to the increase in future ozone level.

## 1. Introduction

Ground-level ozone is a photochemical oxidant regulated under the National Ambient Air Quality Standards (NAAQS) by the U.S. Environmental Protection Agency (EPA). Evidence from epidemiological and toxicological studies has consistently linked ozone exposure to adverse health outcomes, including morbidity and mortality for cardiovascular and respiratory diseases [1–5]. While ozone level has declined due to emission control in many regions of the U.S. over the past two decades, its adverse health effects remain a public health concern [6,7]

Ambient ozone arises mainly as a secondary pollutant formed via reactions between nitrogen oxides and volatile organic compounds in the presence of sunlight. In urban setting, sources of ozone precursors include emission from industrial facilities, power generation, and vehicle exhaust [8]. Future ozone levels are sensitive to climate change because of its formation depends strongly on weather conditions [9]. Examples of weather factors include temperature, wind speed, cloud cover, solar radiation, and atmospheric mixing. Particularly in Eastern U.S., episodes of high ozone concentration are associated with slow moving and high pressure systems characterized by warm temperature, light wind and cloudless skies [8].

There is a growing interest in studying the potential effect of future weather patterns on ozone levels, and its subsequent effects on public health [10,11]. Several studies have reported increases in mortality and hospital admissions attributable to future ozone levels on different spatial scales, ranging from continents [12] to individual U.S. state [13], county [14], and city [15]. While these studies utilize different climate data and employ different analytic approaches, they provide evidence that climate change is likely to increase ozone concentrations, resulting in higher mortality and morbidity [16].

This study contributes to the existing evidence by quantifying the impact of future ozone levels on non-accidental mortality across 19 urban communities in Southeastern United States. This region was selected because it has the greatest increase in 20-year return values of daily maximum temperature in the U.S. [17] and increase in heat-waves according to the Intergovernmental Panel on Climate Change (IPCC) Working Group I Fourth Assessment Report, Chapter 10 [18]. The EPA’s Interim Report of the Global Change Research Program also indicates that this region has high uncertainty in ozone level projections [19].

**Figure 1:**
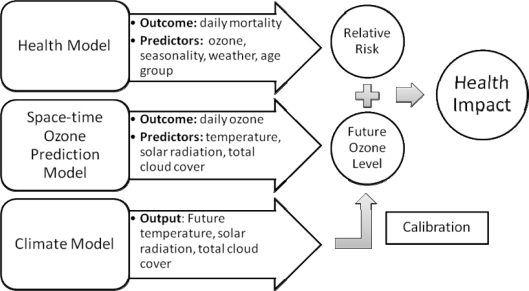
A modeling framework integrating climate model outputs, meteorological observations, and health data to quantify the health impacts of future ozone level.

We also describe a modeling framework that integrates data from (1) climate model outputs, (2) historical air pollution and meteorology measurements, and (3) health surveillance database to quantify the health impacts of future pollution level. Figure 1 illustrates the modeling framework of our approach. In contrast to previous investigations that project future ozone levels by numerical models such as the Community Multiscale Air Quality (CMAQ) modeling system [20], we projected future ozone level via statistical modeling. Specifically, we first constructed a space-time model to describe present-day relationship between maximum daily 8-hour average ozone concentrations and weather variables using recorded monitoring observations. Characterizing the complex relationship between ozone and weather using a statistical model has several advantages. First, it enables us to study potential changes in ozone levels due to climate change by using calibrated weather outputs from regional climate model as predictors. Regional climate models (RCM) are downscaled version of global climate models (GCM) for studying the temporal and spatial evolution of climate based on physical processes. RCM provides finer spatial resolution driven by GCM boundary conditions. Furthermore, projecting future ozone levels through statistical models can reduce computational burden compared to a processed-based numerical modeling approach.

Studies that utilize numerical models for ozone projections also require future weather and emission projections from RCM as inputs. However, since the model outputs are deterministic, quantifying the uncertainty in risk assessment is typically carried out by conducting sensitivity analysis under different future climate scenarios and meteorology simulations. In our approach, projecting future ozone level through a statistical model provides an additional uncertainty measure that can and should be incorporated in estimating attributable deaths. However, similar to previous studies, our approach only considers changes in ozone concentrations due to climate change alone.

Finally, the short-term (acute) adverse effects of ambient ozone on mortality were estimated. Here ambient concentration was viewed as a surrogate for ozone exposure due to outdoor sources. The relative risk of ozone exposure was then used to quantify the health impact of future ozone levels. All estimation was conducted under a Bayesian framework such that uncertainty in each step was incorporated in the final attributable death estimate.

## 2. Methods

### 2.1. Ozone Concentration Prediction Model

The study region (EPA Region 4) consisted of the states Alabama, Florida, Georgia, Kentucky, Mississippi, North Carolina, South Carolina and Tennessee, representing the Southeast United States. We first modeled present-day relationships between observed ambient maximum daily 8-hour average ozone concentrations and three meteorology variables during the year 2000. This metric of daily ozone level was selected because it is used in determining non-attainment status under NAAQS. Specifically, to attain the current ozone standard, the 3-year average of the yearly fourth-highest (99th-quantile) daily maximum 8-hour average ozone concentrations measured at each monitor within an area must not exceed 75 ppb.

Within the study region, ozone concentration measurements were available at 111 sites from the EPA Air Quality System (AQS) monitoring network (http://www.epa.gov/mxplorer/index.htm). We restricted our analysis to the summer months from May to September. EPA’s Air Quality System monitoring network was originally established for regulatory purposes. Since ozone formation requires high temperature and light, most monitors are only operational during the summer months when ozone levels exceeding the NAAQS standards are typically observed. Solar radiation (measured as global horizontal irradiance, GHI) and total cloud cover data were acquired from the National Solar Radiation Data Base (http://rredc.nrel.gov/solar/old_data/nsrdb/). Mean daily temperatures were acquired from the National Oceanic Atmospheric Administration’s National Climatic Data Center (http://www7.ncdc.noaa.gov/CDO/cdo). The average distance between ozone and temperature monitors was 36 km and the average distance between ozone and solar radiation/total cloud cover monitors was 23 km. Since locations of ozone and meteorological variables monitors were not colocated, a closest-distance approach was used to link these observations.

Based on exploratory analyses, we modeled daily ozone concentration as a linear function of temperature, solar radiation, and total cloud cover,

(1) $$Y(s,t)\;=\;X(s,t)\;\beta_{r}+e\;(s,t)$$

where *Y* (*s*,*t*) denotes the maximum 8-hour average ozone concentration in ppb at location (monitor) *s* on day *t; X* (*s*,*t*) denotes the corresponding intercept and weather covariates; parameter *β_r_* denotes the vector of state-specific regression coefficients; and *e* (*s*,*t*) denotes the residual error. We did not observe considerable skewness in the summer ozone measurements and therefore did not transform the response variable by log or square-root. While most of the AQS monitors are located in urban or suburban areas, we did not find these coefficients to be considerably different across monitors of different urbanicity.

To capture spatial-temporal correlation unexplained by our covariates, we assumed the residuals followed a Gaussian process with mean zero and a correlation function that is separable and exponentially decreasing in space and time [17]. Specifically, the covariance between residual errors at monitor location *s_1_* on day *t_1_* and at monitor location *s_2_* on day *t_2_*, has the form

(2) $$\sigma^{2}\text{exp}\;(-\;\rho_{s}^{-1}||s_{1}-s_{2}||)\times \text{exp}\;(-\rho_{t}^{-1}|t_{1}-t_{2}|)$$

where || *s*_1_ − *s*_2_ || denotes the distance between the two locations measured in kilometers after projection from longitude/latitude coordinates. Parameters *ρ_s_* and *ρ_t_* describe, respectively, the rate of exponential decrease in correlation per unite increase in distance (km) and time (day).

Estimation of the unknown parameters was carried out in a Bayesian framework [21] and Markov Chain Monte Carlo (MCMC) techniques were employed to generate samples from the posterior distributions [22,23]. The posterior distribution summarizes information from the observed data and prior knowledge based on the statistical model above. The Bayesian approach enabled us to handle complex space-time models with large dataset while incorporating uncertainty in parameter estimation.

To complete the Bayesian model specification, we assigned the following prior distributions: (1) *ρ_s_* followed a uniform distribution with range 10 to 350 (effective range of 30 to 1050 km); and (2) *ρ_t_* followed a uniform distribution with range 0 to 5 (effective range of 0 to 15 days). The prior distributions for *ρ_s_* and *ρ_t_* were selected based on their corresponding effective ranges. Specifically, the effective range of an exponentially decreasing correlation function represents the separation in days or distance where the correlation reaches 0.05 [24]. The effective ranges for the boundaries of *ρ_s_* and *ρ_t_* prior distributions are given in parentheses above. For example, if *ρ_s_* is equal to 350, the residual correlation between two locations is less than 0.05 beyond 1050 km. The prior distribution of the regression coefficients and residual variance followed proper and non-informative conjugate prior distributions. Prior sensitivity for *ρ_s_* and *ρ_t_* were investigated and did not influence our parameter estimation. Inference was based on 3000 posterior samples with 1000 burn-in samples and all implementation was carried out in R version 2.8.0 [25].

To assess prediction performance, we randomly selected 10 hold-out sites and calculated the root mean-squared error (RMSE) between the true ozone levels and their posterior predictive. We also calculated the percent of time the 95% posterior predictive intervals cover the true ozone values to evaluate model assumptions.

### 2.2. Health Effect Estimation

**Figure 2:**
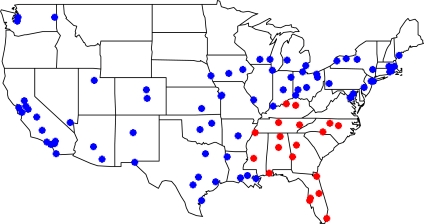
Locations of all NMMAPS communities (blue) used in the health model and the subset within the Southeastern U.S. study region (red).

To estimate the association between acute exposure to ambient ozone and mortality, we utilized data from the National Mortality, Morbidity, and Air Pollution Study (NMMAPS) (http://www.ihapss.jhsph.edu/) [26,27]. The NMMAPS database contains 97 urban communities of which 19 are within our study region as shown in Figure 2. Each community is represented by a single U.S. county, except Atlanta which includes two counties. For each community, daily counts of non-accidental deaths were obtained for the summer months during 1987 to 2000. Daily community-level average ozone concentration, provided by NMMAPS, was calculated by first averaging hourly mean concentration at EPA’s AQS monitors within each community. Then the maximum 8-hour daily average was obtained by selecting the maximum value from an 8-hour running mean of hourly ozone measurements within each day. We also followed EPA’s data completion requirement where at least 75% of the hourly measurements must be available over an 8-hour window.

We carried out a two-stage analysis to combine information across locations following Bell *et al.* [2]. The short-term health effects of ambient air pollution are typically small. In a two-stage analysis, the goal is to combine evidence, borrow information across locations, and potentially enhance statistical power. Unlike a standard meta-analysis, multi-site analysis ensures that the same analytic method is used at each location, minimizing publication/selection bias and allowing better generalizability of the results [28].

For each community separately, relative change in rate of non-accidental mortality associated with ozone levels was estimated via Poisson regression with over-dispersion [29]:

(3) $$\text{log}\;(E[y_{t}^{c}])=\;\beta^{c}x_{t}^{c}\;+\;\gamma^{c}Z_{t}^{c}$$

where *E*( $y_{t}^{c}$) and $x_{t}^{c}$ denote, respectively, the expected number of deaths and the ozone exposure on day *t* in community *c*. Parameter *β^c^* denotes the community-specific relative risks. We examined the effects of same-day (lag 0), 1-day prior (lag 1), and 2-day prior (lag 2) ozone level. We also used an unconstrained distributed lag approach [30] to estimate the overall effect across the three days.

The health model in (3) includes known confounders, $Z_{t}^{c}$, day-of-the-week and age-specific baseline risks (<65, 65–74, >=75 years) and their interactions with calendar date. The effects of (1) same-day and previous-three-day mean temperature, (2) same-day and previous-three-day average dew-point temperature, and (3) long-term trends were controlled for via natural cubic splines with degrees of freedom given by Samet *et al.* [26]. Parameter *γ^c^* denotes the regression coefficients for the confounders.

Finally, county-specific relative risks were pooled under a Bayesian hierarchical model [31]:

(4) $${\hat{\beta }}^{c}∼\;N\;(\beta^{c},{\hat{\nu }}^{c})\;\;\;\;\;\;\;\;\;\beta^{c}\;∼\;N\;(\mu ,\;\tau^{2})$$

where *β̂^c^* denotes the maximum likelihood estimate of the community-specific relative risk and *ν̂^c^* denotes the corresponding estimation error variance. Parameter *τ*^2^ measures the amount of heterogeneity among the true relative risks. The notation *N(a*, *b)* denotes the Normal distribution with mean *a* and variance *b*. The overall average relative risk, *μ* , was estimated using the method of Everson and Morris [32]. We conducted sensitivity analysis by increasing the degrees of freedom of calendar date [33] and found the resulted pooled relative risks to be consistent.

### 2.3. Future Ozone Concentration Prediction

Future daily forecasts of temperature, solar radiation, and cloud-cover were obtained from the North American Regional Climate Change Assessment Program (NARCCAP) as gridded output with a 50km by 50km spatial resolution for the period 2041 to 2050 (http://www.narccap.ucar.edu). The data were generated by the Canadian Regional Climate Model (CRCM) [34] using boundary conditions from the third version of the Coupled Global Climate Model (CGCM3) [35]. Further details of the CRCM can be found at http://www.ec.gc.ca/ccmac-cccma/. The NARCCAPS simulations were conducted under the A2 emissions scenario established by the IPCC [36]. The A2 scenario projects large population increases, high carbon dioxide emissions, and weak environmental concerns. The A2 scenario also describes regionally oriented economic growth with slower and more heterogeneous technological changes.

Daily 8-hour maximum ozone concentrations for the 19 communities were obtained as follows. Biases in climate model outputs when compared to their historical values are well documented [37]. Therefore, future values of temperature, solar radiation, and cloud-cover from climate model were first calibrated before serving as predictors for future ozone level. We assumed a county-specific linear relation between the observed meteorology and those provided by climate model. The intercept and slope for calibration were estimated using data from year 2000 separately for each predictor and for each county. We then treated the fitted value as the calibrated future weather projections. This approach represents a simple form of regression calibration in the measurement error method literature [38]. We treat the model outputs as an error prone version of the unobserved true values and used the observed weather values as validation data.

For each county, future climatic data was linked to the NAARCAPS grid cells within the county. We ignored the spatial change of support problem by treating gridded output as point-level values at the center of each grid cell. The change of support problem occurs when the spatial data are available on different scale [39]. Specifically, here the health outcome is aggregated over a county, while weather and ozone projections are available as average values across 50km by 50km grid cells. Assigning community-average concentration as the average exposure over a grid cell can introduce measurement error. However, empirical evidence has showed that ozone concentration is considerably smooth across the geographic region of a county [8].

Finally 100 samples of the posterior predictive distribution were drawn from the space-time ozone prediction model described in Sahu *et al.* [40]. We assumed the covariance structure of the residual errors for 2041–2050 is identical to the year 2000. Therefore, this prediction approach assumes that the estimated change in ozone level corresponds to the scenario of future temperature, solar radiation, and cloud cover occurring during the year 2000.

### 2.4. Attributable Deaths Calculation

Let *N* be the total number of deaths in year 2000 across the study communities, Δ*x* be the difference in the average ozone level between 2000 and average ozone level for the future period, and *β* be the pooled overall relative risk of mortality per ppb increase in ozone concentration. Assuming the baseline mortality rate and the at-risk population size in 2000, we estimated the change in mortality attributed to future ozone level as *M* = {exp(*β*Δ*x*) −1}× *N*. The standard errors corresponding to Δ*x* and *β* were estimated based on their posterior samples and were assumed independent. Finally, we constructed a 95% confidence interval of *M* by the delta method [41] that combines uncertainty from the ozone projection and the relative risks estimate, while taking into account their non-linear relationship with daily mortality.

## 3. Results

**Figure 3:**
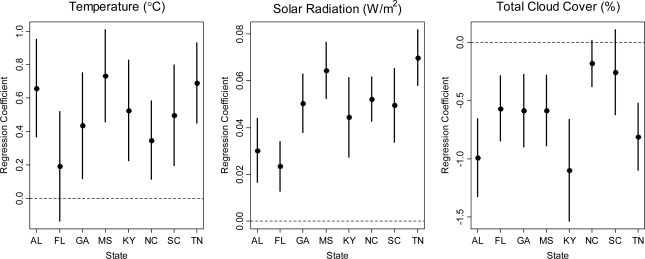
Posterior mean and 95% posterior intervals of state-specific regression coefficients for the model of daily maximum 8-hour ozone concentration and meteorology variables in 2000.

Figure 3 give the parameter estimates and 95% posterior intervals (PI) for the model of daily maximum 8-hour ozone concentration in 2000 as described in Section 2.1. We found that ozone concentration was positively associated with temperature and solar radiation, but negatively associated with total cloud cover. There is also evidence the association between weather variables and ozone level can vary across states. Across 111 monitors, the summer average level of ozone concentration, temperature, solar radiation (GHI), and total cloud-cover was 53.3 ppb, 24.2 °C, 240 W/m^2^, 4.42%, respectively. Therefore total cloud-cover explained considerably less variation in ozone concentration compared to temperature and solar radiation.

We also found significant spatial and temporal dependence in the residuals which had an estimated correlation of 0.58 between consecutive days and a correlation of 0.57 at locations 100 kilometers apart. The corresponding posterior means and 95% posterior intervals for *ρ_t_*, *ρ_s_*, and *σ* are 1.83 (95% PI: 1.76, 1.90), 179 (95% PI: 172, 186), and 10.5 (95% PI: 10.3, 10.8), respectively. Finally, using 10 randomly selected monitors as a validation dataset, the overall root mean-squared error was 7.4 ppb and 94% of the 95% predictive intervals for daily ozone concentration included the true value.

The estimated adverse association between daily mortality per 10 ppb unit increase in maximum 8-hour ozone level on day with lag 0, 1, and 2 were 0.11 (95% PI: 0.00–0.22), 0.23 (95% PI: 0.13–0.33) and 0.11 (95% PI: 0.07–0.16) respectively. The overall (cumulative) relative risk across the three days was 0.26 (95% PI: 0.11–0.41). We carried out a sensitivity analysis by including same-day PM_10_ level in the health model. However, this reduced the sample size considerably for each county. Specifically, during the summer months, ozone measurements were availably every day for most counties; however PM_10_ measurements were typically available only once every sixth day. We did not find evidence that PM_10_ significantly confounds the mortality risk associated with ozone levels due to the lack of data. However, previous analysis of the same population that utilizes the full time series has shown that the county-specific relative risks are robust against PM_10_ adjustment [4]. Therefore we used the unadjusted relative risks for the attributable death calculations.

Across the 19 counties, we found the correlations between observed values and model outputs to be low. The median and inter-quartile range (IQR) of r^2^ across counties, for temperature, solar radiation, and total cloud cover were 0.25 (IQR: 0.08), 0.03 (IQR: 0.02), and 0.01 (IQR: 0.01), respectively. Because of the low correlation between observed and model cloud cover, our calibration algorithm used the 2000 average of 4.42% (the intercept of the linear calibration fit) as the future cloud cover value in the ozone model. This effectively assumes that the average cloud cover in the 2040’s is identical to 2000 due to the lack of information. Despite the low agreement between observed values and model outputs, we found that upon calibration, the statistical model still provides reasonable ozone level prediction for our spatial and temporal scale. Specifically, using calibrated meteorology, we estimated the average ozone level across 19 NMMAPS communities in 2000 to be 51.0 (95% PI: 50.7–51.3) ppb. The true average exposure based on AQS monitors in the communities was 51.0 ppb.

Finally, we used the calibrated future meteorology to estimate average ozone levels over the period 2041–2050 and found an increase of 0.43 (95% PI: 0.14–0.75) ppb across the 19 communities compared to year 2000. This corresponds to a 0.01% increase in mortality rate associated with future ozone level due to climate change alone. The 19 communities had a population of approximately 14.6 million based on the 2000 Census and the baseline mortality rate was 26.9 per 10,000 individuals during May to September per year. Table 1 gives the estimated number of non-accidental deaths associated with this increase in future ozone level across our communities. These estimates were obtained based on a baseline mortality count of 39,514 across the 19 communities in 2000. These NMMAPS communities presented approximately 30 percent of the whole population in the eight states based on 2000 Census.

## 4. Discussion

This paper describes an approach to evaluate the health impacts of future ozone level and presents an application of estimating the number of attributable deaths for the period 2041 to 2050 in Southeastern United States. Our approach differs from previous studies in that ozone projection is obtained through statistical modeling, instead of numerical models. Our modeling framework integrates data from climate model outputs, historical weather and ozone observations, and a health surveillance database. Moreover, all data presented here are publicly available and can be downloaded from online databases, allowing opportunities for future studies and reproducibility.

Our results are comparable to the findings of the previous processed-based numerical modeling study most closely matches our spatial and temporal scale [13]. Tagaris *et al.* reported U.S. state-level change in ozone level between 2050 and 2000. Ozone projections were obtained by CMAQ under the IPCC-A1B scenario of rapid economic and population growth which peak at mid-century. Tagaris *et al.* estimated a 0.71–2.14 ppb increase across southeastern U.S. and the number of excess death for a single year in 2050 ranges from 5 to 70 per state.

Our relative risk estimates are small in magnitude compared to that given in Bell *et al.* [2] due to the different exposure metrics (8-h maximum versus 24-h average) and time scale (summer versus complete year), but are consistent with a similar analysis conducted by Smith *et al.* [42].

In this paper, we propose the use of a statistical model to project future ozone levels due to climate change. The strong relationships between daily ambient ozone concentrations and weather variables are well established. Statistical models have also played an important role in ozone forecasting [43, 44] Moreover, the predictors selected in the statistical model inherently reflect the underlying ozone formation process. For example, daily temperature may serve as a proxy variable for the intensity of UV, and the emission and reaction rate of ozone precursors. Therefore, while our ozone projection model does not arise directly from the complex ozone formation process, we believe it has value in studying climate change and health.

Our ozone projection approach is considerably less computationally intensive compared to process-based numerical modeling. Reducing the computational burden has the advantage that sensitivity analysis can be conducted more easily to assess uncertainty in ozone projection. Specifically, future weather projections based on (1) different emission scenarios or (2) different global/regional climate models, can serve as predictors in the statistical model. While we focused only on Southeastern U.S., the analysis can be extended to additional geographical regions and time periods. Our approach can also be applied to other air pollutants or additional health outcomes such as emergency hospital admissions or number of missed school days for children. Finally, future daily ozone levels obtained from the prediction model can be used to examine additional measures of ozone level indicators. Examples include (1) the National Ambient Air Quality Standards non-attainment status or (2) the number of days where ozone level is *Unhealthy for Sensitive Groups* (Orange) based on the Air Quality Index.

This study has several limitations. First, because our ozone prediction model assumes the regression coefficients to be state-specific, we found the predictive performance of our model to vary across NMMAPS communities. We therefore did not report future death count estimates at the community level. Models that allow for coefficient heterogeneity, for example spatially-varying coefficients [21,45], will enable us to examine health impact of future ozone levels at a finer spatial scale. Sensitivity of the projected health impacts due to model choice and the spatial-temporal scale used for comparison warrants further investigation. For example, our ozone model lacks a separate component for observational measurement error. While this assumption simplifies the Bayesian computation considerably, future studies should evaluate its impact by characterizing this error using data from the AQS.

The two major sources of uncertainty in future ozone projections are (1) changes in emissions of ozone precursors and (2) changes in weather condition. Our approach handles the above two factors similar to studies using process-based numerical model. For example, ozone projection using CMAQ also requires inputs from climate model for future weather conditions and often assumes that the precursor emissions to be unchanged. Since our statistical model is developed using data from a particular reference period (year 2000), our projection also implicitly assumes that the same statistical relationship holds in the future and that the precursor emissions remain unchanged.

In this paper, uncertainty in the calibration of the climate model outputs was not incorporated in the final attributable deaths estimates. However, since the calibration was also accomplished via a statistical model, uncertainty can be propagated by viewing future weather projection as a measurement error in covariate problem. For example, one approach is to simulate predicted weather values and combine the resulting ozone projection estimates by multiple imputation techniques. More sophisticated calibration model that considers spatial/temporal dependence or multiple variables simultaneously should also be explored in future studies.

In this study, we utilized exclusively outputs from the Canadian Regional Climate Model. NARCCAP provides 12 sets of projections for different RCM-GCM combinations. Outputs from regional climate models are known the exhibit bias that varies both spatially and across models [37]. Therefore methodological development in calibrating future meteorology that accounts for this phenomenon may improve future ozone projection. Examples include recent approaches that borrow information across climate models or ensembles [46].

Finally, our health impact analysis does not account for future regulatory policies that control local and regional emissions of ozone precursors; expected changes in population structure, behaviors, and size; or the effects of other pollutants and meteorology. However, while the attributable deaths calculation was not based on future population size, the IPCC scenario used for climate modeling includes population growth. These factors that influence the underlying health status of the population or exposure profiles, especially for the susceptible groups, will result in higher or lower ozone-related deaths than reported here. For example, past studies have identified various effect-modifications of the ozone-mortality relation including central air conditioning usage and unemployment rate [42,47]. Moreover, recent epidemiological evidence suggests that temperature may modify the effects of ozone on mortality [48–50] and several studies have examined the adverse health impacts of future temperature and heat waves [51–55]. Assessing the joint impact of temperature and air pollution due to climate change is an important future research direction.

## Figures and Tables

**Table 1.** Estimated number of non-accidental mortality associated with change in ozone level between 2000 and the period 2041–2050 across 19 urban communities in Southeastern United States based on different exposure lag in days.

Exposure lag	Estimate	95% Posterior Interval
Lag 0	19.1	(−4.5, 42.6)
Lag 1	40.0	(6.15, 73.8)
Lag 2	19.1	(3.63, 34.6)
Lag 0–2	45.2	(3.26, 87.1)
